# Adipocytes and microRNAs Crosstalk: A Key Tile in the Mosaic of Breast Cancer Microenvironment

**DOI:** 10.3390/cancers11101451

**Published:** 2019-09-27

**Authors:** Erika Bandini, Tania Rossi, Giulia Gallerani, Francesco Fabbri

**Affiliations:** Biosciences Laboratory, Istituto Scientifico Romagnolo per lo Studio e la Cura dei Tumori (IRST) IRCCS, 47014 Meldola, Italy; erika.bandini@irst.emr.it (E.B.); giulia.gallerani@irst.emr.it (G.G.); francesco.fabbri@irst.emr.it (F.F.)

**Keywords:** adipocytes, microRNAs, Breast Cancer

## Abstract

Breast cancer (BC) is a disease characterized by a high grade of heterogeneity. Consequently, despite the great achievements obtained in the last decades, most of the current therapeutic regimens still fail. The identification of new molecular mechanisms that will increase the knowledge of all steps of tumor initiation and growth is mandatory in finding new clinical strategies. The BC microenvironment, consisting of endothelial cells, fibroblasts, immune cells and adipocytes, plays an essential role in regulating BC development, and recently it has gained great attention in the scientific community. In particular, adipose tissue is emerging as an important target to investigate among mammary gland components. The mechanisms underlying BC progression driven by adipocytes are predominantly unexplored, especially that involving the switch from normal adipocytes to the so-called cancer-associated adipocytes (CAAs). MicroRNAs (miRNAs), a class of gene expression modulators, have emerged as the regulators of key oncogenes and tumor suppressor genes that affect multiple pathways of the tumor microenvironment and adipose tissue. This review concerns a presentation of the role of adipocytes in breast tissue, and describes the most recent discoveries about the interplay between adipocytes and miRNAs, which collaborate in the arrangement of a pro-inflammatory and cancerous microenvironment, laying the foundations for new concepts in the prevention and treatment of BC.

## 1. Introduction

Breast cancer (BC) is the most common cancer in women, and is still a major health issue, especially because of its high grade of heterogeneity, typically related to chemoresistance, and finally to treatment failure [1,2]. Despite that in the last years a number of findings have been achieved regarding both diagnosis and therapy, BC remains among the most challenging malignancy worldwide. Hence, there is an urgent need to discover new molecules, drug combinations, and innovative therapeutic approaches, that can harness heterogeneity and chemoresistance. Therefore, it is becoming crucial to expand the scenario to the molecular mechanisms underlying the main steps of carcinogenesis, from initiation to promotion and progression. The BC tumor microenvironment (TME) is dominated by stromal cells, such as endothelial cells, fibroblasts, immune cells and adipocytes [3]. Adipose tissue has been recognized as a complex machinery with endocrine and metabolic properties, and emerging evidence indicates the potential for adipocytes to affect tumor biology. The molecular mechanisms determining how adipocytes enhance BC progression is predominantly unexplored, thus adipose tissue is emerging as a target to investigate among microenvironment components. 

Some studies have proposed a correlation between microRNAs (miRNAs), a class of gene expression modulators, and other stromal cells, such as cancer-associated fibroblasts (CAFs) [4,5,6] or endothelial cells [7,8], but not many studies have yet taken into consideration the relationship between miRNAs and the adipocytic component. This review will focus on the presentation of the role of adipocytes in breast tissue, since they are among the main figures in the mammary gland, and will report the most recent discoveries about the interplay between adipose tissue and miRNAs that collaborate in the orchestration of TME and cancer. 

## 2. The Role of Adipocytes in Breast Physiology and Cancer

Tumor microenvironment (TME), a complex setting constituted by different cellular populations and soluble factors, emerged in the last decade as a crucial actor in carcinogenesis, and its contribution has been intensely studied. As reported by Hanahan and Weinberg in 2011, TME is implicated in the so-called “hallmarks of cancer”, among which the sustaining of proliferative signaling, angiogenesis, epithelial-to-mesenchymal transition (EMT), invasion and metastasis [9]. Among cellular components of TME, adipocytes have gained great interest in recent years, since the concept of inert lipid-storage function has been overcome, and metabolic and endocrine functions have been recognized. Indeed, their ability to release substances, among which are interleukins (ILs), tumor necrosis factor alpha (TNFα), leptin, adiponectin, hepatocyte growth factor (HGF) and collagen VI, has been studied [10,11]. However, it has been shown that an unbalanced release of soluble factors is associated with the establishment of a pro-inflammatory TME, especially observed in obese patients, in particular in those affected by BC [12].

**Figure 1:**
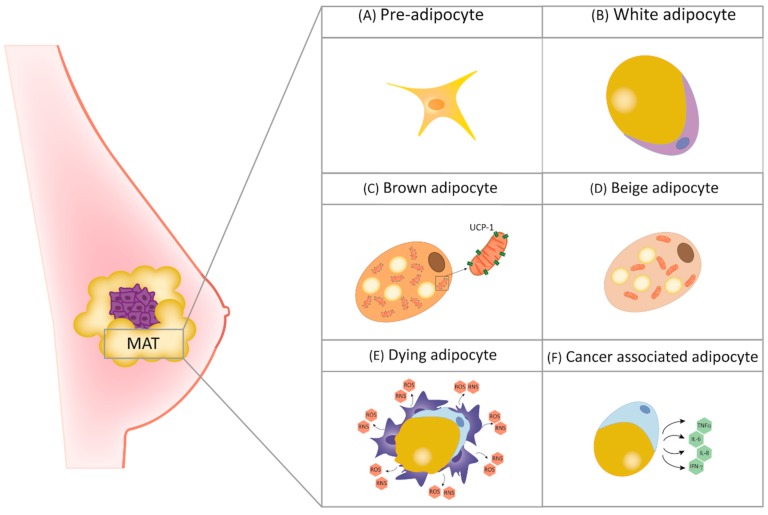
Typical mammary adipose tissue (MAT) cellular composition. (**A**) Pre-adipocytes, immature precursor of mature adipocytes; (**B**) white adipocytes, with lipid-storage and endocrine functions, contain single lipid droplet occupies the largest part of the cytoplasm; (**C**) brown adipocytes, containing several cytoplasmic small fatty droplets and mitochondria, with uncoupling protein 1 (UCP1)-dependent thermoregulatory functions; (**D**) beige adipocytes, with UCP1-independent thermoregulatory functions; (**E**) dying adipocytes, form crown-like structures due to macrophage recruitment, causing the release of reactive nitrogen species (RNS) and reactive oxygen species (ROS); (**F**) cancer-associated adipocytes, with unbalanced production of pro-inflammatory cytokines.

Different types of adipocytes can be distinguished. Although different features and functions may be present, they undergo both physiologic and pathologic-related transdifferentiation, sharing great plasticity [13]. Among them, white adipocytes (WAs), abundant in white adipose tissue (WAT), are spherical cells with triglyceride storage functions in the form of a big lipid droplet occupying the greatest part of the cytoplasm. On the contrary, brown adipocytes (BAs), highly present in brown adipose tissue (BAT), are characterized by thermoregulatory functions and the presence of multiple small lipid droplets in the cytoplasm. Moreover, due to the higher requirement of oxygen than WAs, BAs are characterized by the presence of the high content of mitochondria [14]. Interestingly, BAT could also be originated trough WAT “browning”, a phenomenon mediated by the uncoupling protein 1 (UCP1), which is abundant in BAT and essential for its thermoregulation-related functions. WAT browning has been described as a hallmark of cancer-associated cachexia, a disorder responsible for a high rate of deaths in advanced-stage cancer patients due to skeletal muscle wasting and adipose tissue loss. More recently, other types of adipocytes have been described. For example, the so-called “beige adipocytes” have, like BAs, thermoregulatory functions, even if in a UCP1-independent manner [15]. Being involved in normal mammary gland development, all the mentioned adipocytes are present in tumoral mammary adipose tissue (MAT) (Figure 1) in which cancer-associated adipocytes (CAAs) play an important role in promoting BC progression. Their role in this scenario will be examined better in this review. 

The importance of adipocytes in breast TME arises from the fact that, compared to other organs, its tissue is characterized by a high concentration of fatty cells constituting MAT. Indeed, Lejour showed that the fat content of healthy breasts from individual women ranged from 2 to 78%, with an average of 48% [16]. At a cellular level, MAT shows a high heterogeneity, as it is composed by several cell populations involved in the crosstalk among stroma and breast cells during development, guaranteeing optimal breast morphogenesis [17,18,19]. Besides the high content of mature WAs, undifferentiated fibroblast-like preadipocytes are also present. In particular, preadipocytes derive from the proliferation of stem cells, and give rise to mature adipocytes through differentiation [20]. 

In the last decades, the study of the interaction between BC cells and cellular components of the normal tissue emerged. In this scenario, adipocytes have been widely studied in BC, since as mentioned above, breast tissue is enriched in adipose cells. The role of adipocytes in cancer initiation, progression and resistance to therapy has been described, even if the greatest part of the mechanism needs to be elucidated.

**Figure 2:**
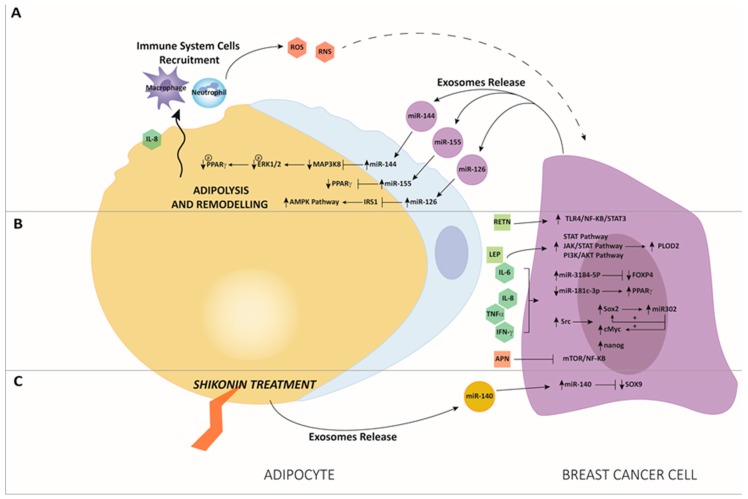
(**A**) After co-culture between adipocytes and breast cancer (BC) cells, high levels of miR-144 and miR-126 were released by exosomes by BC cells. This miRs delivery leads to final adipolysis and remodeling of adipose tissue driven by miR-144 through the downregulation of Mitogen-Activated Protein Kinase Kinase Kinase 8/Mitogen-Activated Protein Kinase 3/1/Peroxisome Proliferator Activated Receptor Gamma (MAPK3/ERK1/2/ pPPARγ) and by miR-126 through the Protein Kinase AMP-Activated Catalytic Subunit Alpha 1 (AMPK)/ autophagy pathway, causing adipocyte-induced tumor growth [77]. BC-derived exosomes after adipocytes interaction carry a higher level of miR-155, which promotes adipocytes differentiation and remodeling in surrounding adipocytes targeting pPPARγ, thus facilitating tumor progression [76]. Increased adipocyte-derived interleukin-8 (IL-8) is responsible for neutrophils and macrophages recruitment, leading to the production of Reactive Oxygen Species (ROS) and Reactive Nitrogen Species (RNS), which act as a threat to tumor initiation. (**B**) Interaction of adipocytes with BC cells increase the release of proinflammatory cytokines and chemokines, leading to an upregulation of miR-3184-5p and downregulation of miR-181c-3p in BC cells, with the modulation of their respective target genes, forkhead box P4 (FOXP4) and PPARα; increased release of adipocyte-derived proinflammatory cytokines and adipokins such as leptin (LEP), IL-6, IL-8 and tumor necrosis factor alpha (TNFα) is responsible for the induction of janus kinase/signal transducers and activators of transcription (JAK/STAT) and through phosphoinositide 3-kinase/protein kinase B (PIK3/AKT) pathways. Resistin (RETN) promotes BC progression through the Toll like receptor 4/nuclear factor kappa-light-chain-enhancer of activated B cells/ signal transducer and activator of transcription 3 (TLR4/ NF-κB /STAT3) axis. As a result, EMT, migration and invasion pathways are enhanced in BC cells [72]. Likewise, after adipocytes and BC cells co-culture, there is an increase of proinflammatory cytokines secretion, with an activation of Src that upregulates SRY-Box transcription factor 2 (SOX2), cMYC and Nanog. SOX2 induces miR-302b transcription that, in turn, further stimulates cMYC and SOX2 expression, increasing the cytokine-induced cancer stem cell-like properties [73]. Conversely, adiponectin (APN) has anti-tumoral properties by inhibiting mammalian target of rapamycin (mTOR) and NF-κB pathways. (**C**) Treatment with the anti-tumor compound shikonin (SK). SK-treated adipocytes release high levels of miR-140, which block tumor progression of co-cultured BC cells targeting SOX9 signaling [74].

For example, it has been shown that the maintenance of a white “behavior” of WAs in the breast seems to be crucial for breast health. Indeed, gene expression profiling of breast adipocytes shows greater browning of mammary fat and greater brown adipocytes-related activity next to malignant breast tumors than in benign breast lesions [21]. More recently, beige and brown adipocytes markers enrichment was found to influence the formation of tumors in mouse models. Furthermore, a study revealed that by inhibiting cyclooxygenase-2 (COX-2), an enzyme involved in inflammation processes and known to induce beige adipocyte formation in mice, reduction of tumor growth was appreciable [22], suggesting the importance of a deeper understanding of beige and brown adipocyte functions. Moreover, the involvement of adipose cells in inflammation has been described as a well-known phenomenon in which the release of pro-inflammatory soluble factors potentially promotes cancer growth, progression and metastatic spread. In fact, the establishment of chronic low-grade inflammation is correlated with increased tumor initiation risk, in a number of cancers, including BC. For example, it has been shown that in metabolic disorders, such as obesity, excessive fat accumulation arises dysfunctional MAT by driving to adipocyte hyperplasia and hypertrophy. As a consequence, increased release of pro-inflammatory soluble factors, such as TNF-α and IL-6, leads to the inflammation of TME [23,24]. Another study linking adipocytes and inflammation reports that dying adipocytes are often encircled by macrophage forming crown-like structures (CLS) that, albeit being normally present in MAT, have been associated with malignant BC also. The presence of CLS seems to be associated, not only with the ability to promote inflammation, but also to induce mutations due to the release of reactive oxygen species (ROS) and reactive nitrogen species (RNS) [25], being potentially involved in tumor initiation (Figure 2A). However, CLS’s role as potential benign breast disease marker has been recently described also [26]. Moreover, obese patients are also characterized by an unbalanced release of adipokines, among which are the pro-tumoral leptin and adiponectin, that conversely is considered the only anti-tumoral and anti-inflammatory adipokine [27]. While leptin accumulation is observed in obesity, where it is thought to contribute to BC development [28], adiponectin and its receptors are less expressed in obese people [29], and such decrease constitutes a possible risk factor for cancer occurrence [30], including those affecting the breast [31]. 

Once the primary BC has been established and malignant cells have invaded MAT, adipocytes switch their phenotype and become cancer-associated adipocytes (CAAs). These activated cells are characterized by increased lipolysis activity and expression of specific markers such as proteinases and the releasing pro-inflammatory soluble factors [32]. Moreover, thanks to the release of miRNAs and extracellular vesicles (EVs), CAAs can fuel the tumor allowing malignant cells to disseminate and protecting it from therapies, sustaining drug resistance. A study conducted by Vazquez Rodriguez et al. highlights the effects of adipocyte-derived IL-8 on BC cells. In particular, up-regulation of IL-8 seems to lead to malignant cell dissemination through different mechanisms, among which the induction of a pro-tumoral phenotype and neutrophils recruitment, whose importance has been recently reported in the early stages of BC metastasis (Figure 2A). Furthermore, low metastatic estrogen receptor-positive BC cells gain metastatic ability through the release of IL-8 by adipocytes, as described in the in vivo metastatic models. Interestingly, IL-8 seems to be a valid therapeutic target, since its reduction induces a significant decrease of metastatic potential [33]. IL-8 has also been recently reported to be a CAA activator, as its ectopic expression has been described to activate tumor-counterpart adipocytes and to enhance their pro-tumorigenic functions in a STAT3-dependent manner. Importantly, IL-8 inhibition repressed CAA malignant functions [34]. IL-6 is another well-known pro-inflammatory cytokine with the ability to mediate the crosstalk among adipocytes and BC cells [35]. Adipocyte-derived IL-6 has also been described to induce EMT through signal transducer and activator of transcription 3 (STAT3) activation, [36] and together with leptin, lead to metastasis by downstream activation of Procollagen-Lysine,2-Oxoglutarate 5-Dioxygenase 2 (PLOD2) through phosphoinositide 3-kinase/protein kinase B (PIK3/AKT) and janus kinase/signal transducers and activators of transcription (JAK/STAT) signaling pathways. In agreement, obese BC patients show higher expression levels of PLOD2 which is associated with poorer prognosis [37]. Furthermore, it has been shown that the adipokine leptin released by adipocytes induces EMT through PI3K/AKT pathway activation, as well as the upregulation of pyruvate kinase M2 (PKM2) is involved in EMT triggering through leptin activity [38]. 

In addition to leptin, resistin, present at high levels in serum from obese people [39], has been shown to be an adipokine with pro-tumoral properties. Indeed, a study conducted by Wang et al. shows that resistin is able to drive to BC progression by inducing stem and mesenchymal features in malignant cells through the Toll like receptor 4/nuclear factor kappa-light-chain-enhancer of activated B cells/STAT3 (TLR4/ NF-κB /STAT3) axis [40]. Adiponectin has also been described to contribute to TME behavior orchestration. In particular, it has been reported that adiponectin can inhibit cell proliferation and stimulate apoptosis in BC cells through the regulation of several pathways, such as mammalian target of rapamycin (mTOR) and NF-κB [41] (Figure 2B). Moreover, low serum adiponectin levels, often encountered in obese patients, were found associated with a higher risk of angiogenesis and metastasis [42].

In TME, adipocytes are also involved in drug resistance, despite the fact that little is known about this event. In vitro coculturing experiments of BC cells with adipocytes conducted by Duong et al. showed that adipocytes and preadipocytes are able to interfere with the antibody-dependent cellular cytotoxicity of trastuzumab by a secretion of soluble factors. In addition, breast tumors grafted next to lipomas showed trastuzumab resistance in mice [43]. Accordingly, tumor-surrounding adipocytes are reported to induce multi-drug resistance through the upregulation of the expression of a transport-associated major vault protein (MVP) conferring a multi-drug resistance phenotype to BC cells. MVP is involved in the efflux of doxorubicin into the extracellular medium through drug-containing vesicles, and its accumulation in the nucleus, in which the drug should bind DNA to carry out its function, is avoided. Interestingly, MVP was more expressed in correspondence of tumor cells present in the tumor periphery rather than in the tumor center. Moreover, cells with a higher expression of MVP seem to disseminate more easily, giving rise to chemo-resistant metastasis [44]. Furthermore, the resistance of malignant cells to doxorubicin-induced apoptosis is conferred by the increased release of the adipokine resistin, an element that is often found at high levels in obese BC patients [45]. Hence, a lower response to therapy in obese patients could be due to insufficient drug dose and altered pharmacokinetics.

## 3. The Interaction between miRNAs and Adipose Tissue in BC

MicroRNAs are the most studied non-coding RNAs, and belong to a large family of short single-strand RNAs (19–24 nucleotides) which participate in many physiological processes, such as cell proliferation, differentiation, death, stress response and inflammatory processes [46,47,48,49]. They act recognizing a “seed-region” in the target messenger RNA (mRNA), consisting of 2–7 nucleotides length, which can be localized in the 3′-untranslated region (UTR) [50], in the 5′-UTR [51] or in the coding region [52]. Their regulatory mechanism is performed through the prevention of the translation of target mRNAs into proteins, but it has also been observed due to an increase of the translation of mRNA, mostly due to an interaction of the miRNA with the 5′-UTR [53,54]. More recently, Fabbri et al. demonstrated for the first time that miRNAs secreted by tumor-derived exosomes (TEX) can act by binding as ligands to the receptors of the TLR family, suggesting a new mechanism of action of miRNAs that become important actors in the tumor microenvironment [55]. Similarly, other studies have contributed to the investigation of miRNAs in the context of tumor communication, leading to a deeper comprehension of processes related to exosomes when released into the extracellular space [56]. Furthermore, a great interest is increasing about the role of extracellular vesicles (EVs) as vehicles for the transfer of functional proteins and nucleic acids, such as mRNAs, miRNAs and long non-coding RNAs [57,58]. Moreover, since exosomes are present in biological fluids, they could become excellent non-invasive markers for cancer diagnosis and for the progression of the disease, associated with the expression of families of ncRNAs [59,60]. It is now certain that inflammatory mechanisms can increase the probability of developing cancer, can promote tumor progression, and support metastatic dissemination. Indeed, inflammatory breast cancer (IBC) is a rare but aggressive form of BC, which represents 2–4% of all BC cases, but with a percentage of 7–10% of BC-related deaths, and with a 20–30% of 10-year overall survival compared to patients with a non-IBC phenotype. Several associated miRNAs were evaluated for their potential applications for the diagnosis and prognosis of IBC, and are becoming interesting to take into consideration [61]. Among the main actors in inflammatory processes, key modulators such as NF-κB, STAT-3, hypoxia-inducible factor 1 (HIF-1), IL-1, IL-6 and TNF, responsible for modulating the expression of tumor-promoting factors, stand out [62]. Researchers have demonstrated that miRNAs can interfere with immune cells acting also as physiological ligands of specific TLRs, triggering the signaling cascade of immune response [63]. Furthermore, adipose tissue has been recognized as a complex machinery with endocrine and metabolic functions [64]. The role of adipocytes in the TME has recently earned interest in different cancer types [65,66,67,68,69] and representing in BC tissue the most abundant stromal cell component, it is becoming crucial to identify their regulatory mechanisms. Carcinoma–adipose stromal cell (ASC) interactions have been considered decisive in the progression of tumor growth, and it appears rather confirmed that paracrine production of cytokines from ASCs stimulates breast carcinoma cell growth [70]. So far, not many studies have proposed a correlation between miRNAs and adipose tissue in BC, but there are increasing findings on their possible role in regulating the adipose microenvironment. Lee et al. explored the role of “cancer-associated adipocytes” (CAAs) in BC tissues, in particular the mechanisms underlying the transition to the inflammatory phenotype CAA. Through co-culture systems between 3T3-L1 adipocytes and BC cells, they reported that the switch to the CAA phenotype was associated with a dysregulation of genes involved in inflammatory pathways such as IL6 and Ptx3 upregulation, together with a pro-proliferative effect of Estrogen Receptor (ER) positive cell lines MCF7 and ZR-75-1. Furthermore, the miRNA array performed on 3T3-L1 cells highlighted an upregulation of mmu-miR-5112, whose target is cytoplasmic polyadenylation element-binding protein 1 (Cpeb1), a negative regulator of IL6 [71]. 

On this trail, through a genome-wide analysis, Rajarajan et al. identified 98 miRNAs as differentially expressed in BC cells after co-culture with mature adipocytes compared to BC cells alone, together with a greater release of proinflammatory cytokines and chemokines by adipocytes, as IL1β, IL6, IL12, TNF-α and interferon-gamma (IFN-γ). Among deregulated miRNAs, sequencing data revealed that miR-3184-5p and miR-181c-3p were respectively the most upregulated and downregulated, with simultaneous downregulation of forkhead box P4 (FOXP4), a gene involved in cancer growth and metastasis, and the upregulation of peroxisome proliferator-activated receptor alpha (PPARα), known to be an oncogene. These genes are targets respectively of miR-3184-5p and miR-181c-3p (Figure 2B). Interestingly, data indicated that the roles of the genes depend on the expression level of their target miRNAs, as well as the microenvironment of the tumor cells. Furthermore, the association of miR-3184-5p inhibitor and miR-181c-3p mimic abolished adipocytes-induced cell proliferation, invasion, and epithelial-mesenchymal transition (EMT) in BC cells, suggesting a strong involvement of the microRNAs in the BC microenvironment regulated by the presence of adipocytes. These findings revealed insights into adipocytes-regulated miRNAs and their mRNAs targets, participating together in the neoplastic transformation of BC cells [72]. Another group analyzed the effects of human adipocyte stem cells (hASC), mature and immature adipocytes co-cultured with BC cells, and reported an increase in the release of proinflammatory cytokines IL6, IL8, IFNγ-IP10, C-C Motif Chemokine Ligand 2 (CCL2) and CCL5 by BC cells, especially after interaction with immature adipocytes. The effect of this interaction resulted in the activation of Src, and thus promoting Sox2, c-Myc and Nanog upregulation. Furthermore, they established a feed-forward mechanism driven by Src that upregulates SRY-Box transcription factor 2 (SOX2), which consecutively induces miR-302b. This microRNA has been recently associated with an expression of stem cell markers, nodal metastasis and poor patient outcome. MiR-302b induction is in turn responsible for the maintenance of c-MYC and SOX2 expression (Figure 2B), leading to the further enhancement of cytokine-induced cancer stem cell-like properties [73]. Gernapudi et al. determined a crucial miR-140/SOX2/SOX9 axis that modulates differentiation, stemness and migration related to TME. They found that in exosomes secreted from pre-adipocytes, miR-140 was implicated in affecting the surrounding BC cells. Starting from the hypothesis that the interaction between adipocytes and BC cells promotes tumor aggressiveness and invasion, they described an inhibition of pre-adipocytes differentiation following a 3T3L1 pre-adipocytes treatment with the antitumor shikonin compound (SK). Since it has already been described that the Sox9 gene is activated in ductal carcinoma in situ (DCIS) stem-like cells, and that miR-140 is a Sox9 regulator, frequently silenced in DCIS and activated after treatment with chemopreventive compounds, they tested if the SOX9/miR-140 pathway could be involved in SK capacity to repress pre-adipocyte differentiation. Results showed that upon the SK treatment of 3T3L1 cells, SOX9 expression significantly decreased together with an increase in miR-140 induction. Furthermore, they showed that exosomes derived from 3T3L1 cells carry detectable levels of SOX9 protein, and that this phenomenon was abrogated after SK addition, along with an increase of miR-140 levels. To confirm miR-140 implication through co-culture systems, they proved that SK-treated pre-adipocytes were able to release exosomes carrying a high level of miR-140, which can affect nearby DCIS cells through targeting the SOX9 pathway (Figure 2C). These data contributed to reinforce the hypothesis of inhibiting breast tumorigenesis by targeting pre-adipocyte and miRNAs signaling in the context of TME [74]. miR-155 is a well characterized oncomiR frequently overexpressed in several cancer types, besides representing one of the first characterized exosomal microRNAs, acting like a cargo shuttled between cancer cells and the tumor microenvironment [75]. Curiously, a study reported that Peroxisome Proliferator Activated Receptor Gamma (PPARγ), involved in lipid accumulation, is a direct target of miR-155. More in detail, it was found that PPARγ levels decreased in adipocytes after co-culture with BC cells and in adipocytes overexpressing miR-155, while PPARγ expression levels were re-established after miR-155 silencing in cultured BC cells. Furthermore, BC-derived exosomes were isolated, and a higher level of mR-155 was detected after adipocyte influence. Likewise, a high level of miR-155 was revealed in cultivated adipocytes. It was established that the energy metabolism of adipocytes was remodeled and was regulated by the exo-miR-155 through the downregulation of PPARγ gene (Figure 2A). 

These results show that cancer cell-secreted miR-155 promotes differentiation and alters the metabolism of surrounding adipocytes by downregulating PPARγ expression, accelerating a cancer-lipolytic process that has been described to be a phenomenon associated with tumor progression [76]. In order to investigate the function of adipocytes in BC progression, Wu et al. reported that after co-culture with BC cells, mature adipocytes appeared to upregulate their beige/brown composition and appeared to undergo a lipolytic process, with a release of metabolites, including free fatty acids, pyruvate, lactate and ketones. Likewise, BC cells exhibited metabolic adaptation and an aggressive phenotype. They found that miR-144 and miR-126 were carried at a high level by exosomes released after tumor-adipocyte interaction (Figure 2A), and that these microRNAs were responsible for the reprogramming of the energy metabolism to facilitate tumor progression. In particular, miR-144 was found to promote beige/brown adipocyte differentiation by downregulating the Mitogen-Activated Protein Kinase Kinase Kinase 8/Mitogen-Activated Protein Kinase 3/1/PPARγ axis (MAP3K8/ERK1/2/PPARγ), while exosomal miRNA-126 interfered with metabolism targeting insulin receptor substrate 1 (IRS1), activating the Protein Kinase AMP-Activated Catalytic Subunit Alpha 1 (AMPK)/autophagy pathway and stabilizing HIF1α expression in adipocytes [77]. In addition to the deepening of the role of adipocytes in BC, in recent years much attention has been paid to the study of adipose tissue, especially in subjects with metabolic dysfunctions, including obesity-related inflammation. This last anomaly is strongly related to the secretion of hormones, adipocytokines, fatty acids and microRNAs also. Indeed, in the last years antiobesity drugs such as metformin have been investigated in preclinical models and clinical trials for their efficacy in improving clinical outcomes of BC patients [78]. Obesity has been progressively recognized as a risk factor for BC development, but the molecular basis of obesity-related breast tumorigenesis is still elusive. A study reported that obesity decreases the level of the tumor suppressor p16INK4A protein in breast adipocytes, characterized by pro-tumorigenic potential both in vitro and in tumor xenografts in comparison to mature adipocytes from lean women, leading to the EMT process of in breast ductal epithelial cells. Intriguingly, downregulation of p16INK4A increased the release of several adipokines, including leptin, and activated breast adipocytes from lean women. Therefore, like the breast adipocytes of obese women, p16-defective adipocytes induced EMT in normal primary breast luminal cells in a leptin-dependent manner, and enhanced tumor growth. Interestingly, p16INK4A was observed to negatively regulate leptin at the mRNA level through miR-141 and 146b-5p. The study presented the indication that the pro-carcinogenic paracrine effects of p16-deficient adipocytes are leptin dependent, and that p16 negatively regulates the expression/secretion of leptin through miR-141 and miR-146b-5p action. Therefore, the study of the p16 pathway and its downstream target tumor suppressor miRNAs show how these miRNAs can be used as molecular biomarkers for obesity and obesity-related pro-carcinogenic effects in BC [79]. Wolfson et al. utilized mouse models to induce obesity and then investigated the impact on myofibroblast differentiation in the mammary stromal microenvironment. They proved an association between induced obesity and myofibroblast differentiation caused by the decrease of miR-140 expression in mammary adipose tissue. In particular, increased transforming growth factor β1 (TGF-β1) in mammary adipose tissue in obese mice stimulates SMAD family member 3 (SMAD3) signaling, leading to the binding of phospho-SMAD3 to the miR-140 locus, and preventing miR-140 transcription. This causes the inhibition of SMAD3 targeting and degradation by miR-140, resulting in amplified TGF-β1/SMAD3 signaling and miR-140 downregulation-dependent myofibroblast differentiation, with an impact on the stemness and proliferation of normal ductal epithelial cells in early-stage of BC invasion. In light of this study, miR-140 may become a novel target to evaluate in the context of a high-fat mammary microenvironment [80].

## 4. Conclusions

Tumor stroma and its cellular components, such as fibroblasts, endothelial cells, immune cells and adipocytes, are known to influence tumorigenesis as well as cancer progression and therapy resistance. Therefore, TME with pro-tumorigenic behavior has often been suggested as a potential therapy target together with cancer cells. For example, in the latest years, cancer-associated fibroblasts (CAFs) emerged as promising targets, since through the release of growth factors and cytokines they participate in the orchestration of the malignant microenvironment [81,82].

Adipocytes, another abundant population of TME so far pretty well ignored, have proven to be crucial in mammary development as well as breast carcinogenesis, progression and chemoresistance. Moreover, adipocytes have been intensively investigated in obese patients, due to their accumulation in adipose tissue. Indeed, hyperplasia and hypertrophy of adipose tissue, that are frequently observed events in obesity, lead to an unbalanced release of adipokines and other pro-inflammatory cytokines that orchestrate the crosstalk among cancerous cells and TME. For this reason, CAAs targeting may become another pivotal therapeutic strategy. Regulation of adipocytes by miRNAs is gaining interest as a novel interaction to explore. It can contribute to a wider comprehension of the mechanisms that regulate the mammary gland TME, especially during inflammatory processes and tumorigenesis. Actually, in vitro studies suggest that miRNAs from adipocytes give rise to a niche from which, under unbalanced conditions, a neoplastic transformation of breast cells may start. In turn, CAAs and adipocyte-secreted factors induce the production of pro-inflammatory cytokines and chemokines, leading to the accentuation of the BC phenotype through the proliferation, migration, invasion and induction of drug resistance [43,44,83]. All of the herein presented aspects, inflammation, microenvironment, adipocytes and miRNAs, are strictly interlinked and imbricated, creating a central network that could pave the way to tumor rise and progression. Consequently, it is not surprising that the study of the interaction between microRNAs and adipocytes in BC can become a new branch to study, in order to find a new way to exploit these small molecules as possible single therapeutics, or in combination with conventional therapies. The studies presented in this review provide insights into adipocyte-regulated miRNAs and their mRNA targets, proposing a novel preclinical rationale to investigate novel inhibitors for BC therapies. Awareness about the nature of this cross-talk will allow for improved therapeutics that simultaneously target multiple factors of the TME, increasing the chance of favorable patient outcomes.
